# Metformin in obesity, cancer and aging: addressing controversies

**DOI:** 10.18632/aging.100455

**Published:** 2012-04-30

**Authors:** Lev M. Berstein

**Affiliations:** Lab. Oncoendocrinology, N.N.Petrov Research Institute of Oncology, St. Petersburg, Russia 197758

**Keywords:** metformin, obesity, cancer, aging, modifying factors

## Abstract

Metformin, an oral anti-diabetic drug, is being considered increasingly for treatment and prevention of cancer, obesity as well as for the extension of healthy lifespan. Gradually accumulating discrepancies about its effect on cancer and obesity can be explained by the shortage of randomized clinical trials, differences between control groups (reference points), gender- and age-associated effects and pharmacogenetic factors. Studies of the potential antiaging effects of antidiabetic biguanides, such as metformin, are still experimental for obvious reasons and their results are currently ambiguous. Here we discuss whether the discrepancies in different studies are merely methodological or inherently related to individual differences in responsiveness to the drug.

The epidemics of obesity and diabetes, which has been the matter of major concern for endocrinologists over the last two decades, has lead to an apparently unexpected, yet actually long foretold [1-3] result: the concern spread not only to cardiologists but, also, to oncologists and specialists engaged in tackling of more general problems, including aging and age-related pathology [4-7]. The wave of interest, with periodical decays and increasing surges, was associated with the attempts to use antidiabetic biguanides to control body weight and tumor growth [8-13]. Another facet of the situation is that almost 45 years ago these drugs were suggested to promote longevity [14]. Over the last years, the expanding bodies of relevant evidence, which mainly related to metformin, started to merge and occupy increasing place in current literature. The objective of the present essay is to attract more attention to accumulating inconsistencies. The first two sections of the essay, which are related to *obesity* and *cancer*, are based mostly on clinical data. The third section, which is related to aging or, rather, *antiaging*, is based predominately on experimental evidence obtained in rodents. Clearly, obesity and cancer have numerous interrelationships with aging (see details in [3, 15, 16]); however, we will separate these aspects for the sake of clarity in discussing the relevant effects of metformin.

## Metformin and obesity

Weight-reducing effects of biguanides may explain in part their antitumor activity [13, 17]. Obesity is associated with increased mortality and, hence, decreased lifespan [2, 4]. Therefore, first of all, we will discuss whether biguanides reduce weight and affect body composition (fat vs. lean mass and visceral vs. subcutaneous fat). Two meta-analyses published in 2005 and 2011 summarized the use of metformin to treat adults having excessive body mass (fat) and showed that, among more than fifty potentially relevant studies, only less than ten satisfied all criteria. Only two or three of the latter confirmed that metformin had moderate weight-reducing effects, which, however, were inferior to the effects of behavioral interventions and gastrointestinal fat absorption inhibitor orlistat [18, 19]. Nevertheless, more scrutiny in treating the data accumulated so far, a part of which reproduces earlier results, suggests that it is reasonable to dissect the evidence into several subsections.

### Obesity in type 2 diabetes mellitus

For obese diabetics and people at risk of diabetes, metformin remains a treatment able to moderately reduce body weight (by 5% on average). This is believed to be an additional benefit in treating diabetes and is suggested to be caused by reducing of insulin resistance and hyperinsulinemia rather than by anorexic or other effects [20, 21].

### Polycystic ovary syndrome (PCOS) in women with excess body weight

Only in a part of the studies in PCOS patients with obesity, metformin treatment without behavioral interventions decreased body mass index (BMI). It is not clear whether this can be explained by metformin underdosage [22], although a recent meta-analysis indicates that both drug dose and duration of treatment are highly relevant [23].

### Age, genetic factors and oxidative stress

According to a recent randomised study [24], confirmed by other publications [25], children and adolescents may be more responsive to metformin-induced weight reduction compared to adults. This is reflected by body weight dynamics and by changes in the waist/hip ratio and fat deposits. Metformin has been shown to upregulate the expression of the organic cation transporter OCT1 in adipose tissue. Therefore, OCT1 may be a potential marker of metformin accumulation in the tissue [26]. On the other hand, bearing of one of the polymorphisms of the neuron growth regulator NEGR1 predicts the magnitude of the weight-reducing effect of metformin and, ironically, the propensity to restore body weight after the discontinuation of treatment with metformin [27]. The significance of oxidative stress also is difficult to characterize. This process is clearly increased in the adipose tissue in obese patients [28], and although metformin is known to have an antioxidant activity, especially in diabetes [29], this drug can enhance free radical generation in differentiating adipocytes [30].

### Metformin and the heterogeneity of obesity

Possible associations between differences in metabolic phenotypes could account for differences in metformin action. The list of conditions is long and includes among others such varieties as ‘standard’ obesity, metabolically healthy obese state (MHO), sarcopenic obesity, and metabolically obese normal weight state (MONW) (see [31]). The incidences of major non-infectious age-dependent diseases, the ways of treating them, and treatment outcomes, especially at advanced ages, are important as well known from both, basic science and, especially, applied science standpoints. With regard to treatment, it should be noted that, although there are still many proponents of the idea that obesity should be treated irrespective of its type, it is also suggested that such a straightforward approach may be inadequate, in particular, when applied to MHO [32]. There is still no evidence about metformin use with regards to the some aforementioned subtypes of obesity, in contrast to numerous studies of its use in the visceral/abdominal obesity (e.g., see [33] and many others). The sarcopenic obesity, i.e. body fat gain associated with skeletal muscle loss, appears in cancer patients to lead to poorer outcomes of endocrine therapy and chemotherapy, treatments that tend to increase body fat by themselves.

### Metformin and body weight in cancer patients

This issue is addressed implicitly in many studies of polycystic ovary syndrome (see above), which is believed to be a risk factor of several hormone dependent malignancies, first of all, endometrial cancer. Several relevant observations relate to prostate cancer patients exposed to androgen deprivation, which is associated often with insulin resistance and other manifestations of the metabolic syndrome, including body weight gain. In one study of such patients, metformin treatment during 6 months was associated with decreased BMI and waist circumference [34]. The important question of the influence of body weight and body fat on the ability of metformin to affect cancer incidence in diabetic patients (see the next section) is unanswered as of yet, although BMI as potential risk modulator was taken in consideration in some of such studies, e.g. [35].

### Cancer incidence in diabetic patients treated with metformin

As noted earlier [36] based on evidence accumulated by mid-2010ies, the emerging picture is rather vague. In particular, metformin effects are rather variable with regard to specific cancer locations, except for two notable cancers where the incidence decreased with metformin (colon cancer and hepatoblastoma). These data suggest that the possible anticancer effects of metformin are tissue-specific. It was reasoned by some authors that it is not that metformin is ‘thus good’, but the reference therapies, including sulfonylureas and insulin, may be ‘thus bad’ [35, 36]. Also, no data were available to judge whether glucose intolerance reduction/compensation by biguanides is important for the total and site-specific cancer incidence shifts.

Much of relevant evidence was gained over the last two years. The main findings may be categorized according to the following subtitles:

#### Cancer location

Reduced colon cancer incidence in diabetic patients treated with metformin was confirmed by meta-analysis of 5 observational studies where total odds ratio (OR) for treated vs. untreated subjects was 0.68 [37]; however, this conclusion was questioned later [38]. Some controversy remains in regards to prostate cancer because in a case-control study of diabetics treated with metformin the OR for prostate cancer was found to increase to 1.23 [39]. In a study based on data obtained from Danish Cancer Register, the OR for breast cancer incidence was found to decrease to 0.77 upon the use of metformin in patients with type 2 diabetes [40]. Despite this positive result, the situation with breast cancer is somewhat debatable, in particular, because several known molecular-biological subtypes of this tumor are significantly different in their risk factors and responses to therapy. Accordingly, some studies found no association between metformin therapy and breast cancer incidence in diabetic women [41, 42]. In a recent study performed in Taiwan, metformin intake by diabetics was found to be associated with reduced risks of colorectal carcinomas (the effect was more expressed in women) and liver cancer (only in men) but not of oesophagus, stomach and pancreas cancer [43].

#### Study design

The aforementioned study from Taiwan [43] may be referred to as prospective, since study subjects, which were recruited in 2000, where followed for 7 to 8 years. Unfortunately, prospective studies related to the ‘metformin-diabetes-cancer risk problem’ are rare and are not designed specifically to tackle this problem. Available publications are largely based on observational or retrospective case-control studies or, rarely, cohort follow-up [35]. To the best of our knowledge, there is only one meta-analysis of randomized controlled trials (RCTs) that was reported at the Annual American Diabetes Association Conference in June 2011 [44] and cited in the editorial article [45]. The analysis was based on 4 published trials and 3 datasets obtained directly from Principal Investigators. Inclusion criteria for the trials stipulated that there must be ≥ 500 diabetes mellitus patients followed for ≥ 1 year. Output parameters were cancer incidence and all-cause mortality. The OR for cancer risks upon metformin intake vs. other therapies varied from 1.03 to 1.47 arguing against the ability of metformin to reduce cancer incidence in diabetic patients [44]. It cannot be excluded, however, that the above meta-analysis included the data of an independently published study [46] based on ADOPT and RECORD RCTs that did not show differences between metformin and roziglitazone with regard to their effects on cancer risk, but did demonstrate and confirm that metformin affords some benefit compared with sulfonylurea. To tell the truth, both these trials mentioned in the paper of P.D. Home et al. [46] were not designed specifically to address cancer incidence (as well as many previous observational studies that suggested a reduction of cancer risk by metformin therapy).

#### Reference points

The importance of ‘reference therapy’ or its absence (placebo) for judging the effects of metformin follows from the preceding paragraph and was mentioned above. Studies where data on metformin-treated diabetes were compared with untreated diabetes are few for obvious ethical and morbidity reasons. One such study is the aforementioned Taiwanese study [43] where total cancer incidence per 10 000 person×years were reported for “no diabetes” (46.0), “diabetes treated with metformin” (44.8), “diabetes treated with other antidiabetic therapies without metformin” (91.7), and “diabetes without any treatment” (97.6). The last two figures are very close suggesting that differences between metformin and other drugs may be associated with the adverse effects of the latter. Such proposals, in particular related to sulfonylurea derivatives, have been put forward several times before and are exemplified by the recent quotation: “However, whether this should indeed be seen as a decreased risk of cancer for the use of metformin compared with the use of sulfonylurea derivatives or as an increased risk of cancer for the use of sulfonylurea derivatives compared with the use of metformin remains to be elucidated” [47].

It should be noted that when cancer-related mortality, rather than cancer risk, is assessed in diabetics (once again, at ages mainly above 50), the belief that their survival is better in metformin-treated groups is not always confirmed for prostate and breast cancer [48, 49], although one study does suggest better survival for these cancers as well [50]. Metformin was found to be relatively more beneficial with regard to colon and ovarian cancer survival in diabetics [51, 52]. However, in these studies, metformin was compared with other antidiabetic drugs, whose less favorable effects cannot be ruled out.

### Effectiveness of biguanides in cancer patients without diabetes

#### Past- vs. present-time evidence

The currently discussed prospects for using antidiabetic biguanides as potential antitumor drugs in cancer patients without diabetes [13, 53] attracted a great deal of attention in the past. In particular, it was shown in studies initiated by Prof. V.M. Dilman more than 40 years ago that the inclusion of phenformin as a long-term adjuvant modality into programs of metabolic rehabilitation of postoperative colorectal and breast cancer patients was associated with increased total and relapse-free survival [10, 54, 55]. In recent study, metformin was administered at a dose of 500 to 1000 mg daily for 2 to 4 weeks before surgery (that is, in a neoadjuvant manner) to breast cancer patients. Most of the patients, whose mean age was 59.9 years, were postmenopausal. This treatment was associated with decreases in the mean mitotic index and signaling pathway activity in tumor tissues, although blood insulin was not changed [56]. In another publication, which is cited highly now, it was reported that the daily low doses (250 mg) of metformin used in patients without diabetic manifestations decreased the number of aberrant crypt foci in the rectum and cell proliferation rate in colon epithelium as early as one month after the onset of the treatment [57]. At the same time, according to our data, in a metabolically similar group of postmenopausal endometrial cancer patients treated with metformin (1.5 g/day) in a neoadjuvant mode for 5.3±0.7 weeks, endometrial thickness (sonographic M-signal) did not decrease, a finding that is in contrast to the decreases observed earlier with aromatase inhibitors, while the expression of the cell proliferation marker Ki-67 decreased in only one third of the patients [58].

#### Clinical trials

Randomized clinical trials are now underway in several countries, mostly under auspices of the National Cancer Institute (http://www.clinicaltrials.gov/), to clarify the issue of metformin usability in nondiabetic patients. However, one of the initiators of these trials, Prof. Pamela Goodwin (University of Toronto, Canada), who, in particular, is directing a large multicenter, 5 year study of adjuvant metformin in nondiabetic breast cancer patients, is rather sober in her recent comment published in Science [59]: “We anticipate that it will take another 2 years to enroll everyone and maybe 2 to 3 years after that before we have results. All the preclinical and epidemiological evidence is pretty consistent and compelling, but all it's done is help us form a hypothesis… Until then, the best we can say is that metformin *may* be beneficial for cancer. We need to proceed from here very, very carefully.”

#### Resistance and responsiveness to metformin, and its tolerability and pharmacogenetics

The aspects listed in the subtitle are relevant directly to metformin use in obesity clinics (as was mentioned already above with regard to resistance), oncology [36, 60] and, undoubtedly, aging research (see [7] and below) and therefore need further scrutiny. Unresponsiveness to metformin, which was displayed by a number of normal and transformed cell lines, is probably caused by specific features of mitochondrial function as they relate to apoptosis [61]. In the field of pharmacogenetics, the relatively long known polymorphism of the organic cation transporter OCT1 [62] has been added to the ever increasing number of other markers associated with differences in the metabolism of biguanides and, thereby, in their effects [63, 64]. It is well known that, mainly because of gastrointestinal discomfort, metformin treatment is cancelled or interrupted in every fifth to sixth diabetic patient, and the rate of such adverse effects is increased in elderly subjects [65]. Such effects also are observed in nondiabetic cancer patients treated with metformin [56]. According to the latest Cochrane Collaboration estimates, the risk of lactic acidosis resulting from metformin intake (4-5 cases per 100000 subjects×years) is lower than previously thought [66]. In this regard, metformin is 7-10 times better than phenformin. Moreover, there is no evidence that antidiabetic biguanides can induce lactic acidosis in nondiabetics, even at older or advanced ages [13, 56]; therefore, the gastrointestinal side effects, especially in the elderly, seem to be the primary concern associated with metformin usage.

## Metformin in the antiaging research agenda

Potential antiaging drugs are expected to prevent or eliminate age-related diseases [7]. Evidence that metformin is more beneficial that other antidiabetic drugs in reducing all-cause mortality and, therefore, increasing life expectancy in diabetic patients was presented earlier. This important feature is believed to be associated with the ability of metformin to influence the rate of macrovascular complications of diabetes [67, 68] rather than the basic mechanisms of aging. Such mechanisms as potential targets of metformin are under increasing scrutiny in the recent years. Among proximal targets under discussion are those involved in insulin resistance, insulin/IFG-1 system, and fatty acid oxidation and utilization [7, 69-71], which were considered earlier with regard to the antiaging effects of phenformin [3, 14, 72]. Among the most discussed targets of metformin are AMPK activity and AMP-related signaling, glycation reactions and glycation end-products, mitochondrial membranes, reactive oxygen species generation, epigenetic mechanisms, pluripotent stem cells, cell proliferative senescence and mTOR pathway [7, 71, 73-77]. Without digging into all possible mechanistic details, the only endpoints used to assess metformin as an antiaging agent will be considered below.

Metformin has been shown to slow-down lipofuscin accumulation, enhance locomotor activity and increase mean lifespan in Caenorhabditis elegans nematodes in a dose-dependent manner within concentration range of 1 to 50 mM in culture medium [78]. In R6/2 mice, used to model Huntington's disease, metformin increased the lifespan of males, but not of females at a concentration of 2 mg/mL in drinking water but not at 5 mg/mL [79]. However, in order to differentiate changes in rodent lifespan resulting from influences on the basic mechanisms of aging rather than on specific disease-related mortality, it is more appropriate, according to S.R.Spindler [80], to use genetically heterogeneous long-lived healthy populations, because short-lived or weakened animals have not been shown to predict longevity effects observed in long-lived ones. It is also mandatory to report the data with regards to monitored food consumption and body weight, thereby excluding the potential effects of caloric restriction; more than that, a positive control (e.g., a calorically restricted group) is highly desirable too [80]. Of note, rodent species, such as mice and rats, as well as nematodes and fruit flies, originated as a consequence of r-selection with an emphasis on a high growth rate resulting in numerous offspring, each of which has a relatively low probability of surviving to adulthood. In contrast to that, higher primate species including humans were molded in evolution primarily by K-selection resulting from living in crowded niches and having fewer offspring, each of which has a relatively high probability of surviving to adulthood [81, 82]. Nevertheless, for a number of practical reasons, properly chosen rodents remain the best choice for the selection of lifespan expanding drugs [80].

The aforesaid is of importance in considering experimental data about metformin, which are presented in Table 1. Most experiments were performed by the group lead by Prof. V.N. Anisimov [70, 83-88] with involvement, in some cases, of our laboratory. It is necessary to note, that unfortunately not all of these experiments fully satisfy the above requirements [80]. For example, the transgenic HER-2/neu mice are short-living and die mainly of mammary carcinomas. In some experiments, metformin-treated animals exhibited changes in food-consumption and body weight (see Table 1 and [83-85, 87-88]). Nevertheless, their representation, supplemented by the results reported by D.L. Smith et al. [89], helps to see the whole picture. Certain sex-related differences in effects observed in mice [87] were discussed earlier [90]. Noteworthy is that metformin tends to be more efficient upon earlier onset of its application ([88] and Table 1). This data corroborates previous experimental observations related to newborn macrosomy [91] and suggests that early-onset usage of biguanides, including during pregnancy and even preceding pregnancy, may be more beneficial than the late-onset, notwithstanding all difficulties in the practical realization of such recommendations.

**Table 1:** Data on lifespan in rodents receiving metformin

Species, gender and strain	Drug administration mode and the age of its onset	Food consumption vs. control	Body weight vs. control	Lifespan, days (control/experiment, % change)			Ref.
				Mean	Maximal	Last 10% survivors	
Mice, F Her-2/neu transgenic (FVB/N)	Drinking water, 100 mg/kg, 2 months	Decrease on months 4 & 6 (p <0.05)	No changes	264±4/285±5 +8.0%	311/340 +16.2%	297±7/336±3 +13.1%* p<0.05	[83]
Mice, F Her-2/neu transgenic (FVB/N)	Drinking water, 100 mg/kg, 2 months	Decrease on months 7 & 8	No changes	285±12/304±10 +6.7% p for log-rank test 0.21	C 396/359 −9.3%	396/352 −11.1%* p<0.001	[84]#
Mice, F SHR	Drinking water, 100 mg/kg, 3 months	Increase on months 12-16 (p<0.05) and decrease on month 22 (p<0.01)	Tends to decrease after 20 months	388±29/535±32 +37.9%* p<0.01	814/898 +10.3%	727±23/878±7 +20.8%	[85]
Mice, F SHR	Drinking water, 100 mg/kg 2 months	No difference	No changes	559±22/583±27 +4.1%	941/972 +3.3%	892±12/897±28 +0.6%	[86]##
Mice, F SHR	Drinking water, 100 mg/kg 3 months 9 months 15 months	No difference	No changes No changes Decreases after 20 months	511±20/583±27 +14.1%, p= 0.17 583±18/619±20 +6.2% 668±16/647±21 −4.2%	941/972 +3.3% 941/855 −9.1% 941/966 +2.7%	881±13/897±28 +2.0% 892±12/820±14 −8.8% 913±9/892±47 −2.3%	[88]##
Mice, F 129/Sv	Drinking water, 100 mg/kg 3 months	Decrease on months 15-21	Decreases after 25 months	706±21/742±16 +5.1%	930/966 +3.9%	910±9/913±19 +0.3%	[87]
Mice, M 129/Sv	Drinking water, 100 mg/kg 3 months	Decrease on months 15-21	Decreases after 22 months	662±28/573±27 −13.4%*	1029/1044 +1.5%	951±32/931±30 −2.1%	[87]
Rats, M Fisher F344	Chow, 300 mg/kg 6 months	No difference	Decreases at 48-74 months	796±170/ 815±186 +2.4%	1065/1062 −0.3%	1039±30/1061±3 +2.1%	[89]

* Notes: differences are statistically significant

# Data in [84] are not fully consistent with [83]

## Data in [86] and partly in [88] do not reproduce data in [85]; the mean lifespan in control was much shorter in [85] than in [86, 88]

On the whole, the data collected till present unfortunately are not always reproduced in the same experimental settings (Table 1), and demonstrate mixed results as has been already pointed out (see [89, 93] and in part [71, 92]). This conclusion relates as well to slowing down of aging rate assessed by the Gompertz model [83] since shifts of α parameter value in this model into the favorable side were not found in all the experiments mentioned above.

## Conclusions

The main and uniform conclusion presented herein is that metformin acts ‘selectively’ and its effects on obesity, cancer, and lifespan experiments vary depending on gender [90], age [24, 25, 51, 88] and other factors, including tumor location (tissue specificity). It has been pointed out earlier [36, 55] that, in order to make use of the potential antitumor and/or weight-reducing activities of metformin, it is necessary to consider its direct and indirect effects, the presence or absence of glucose intolerance, and impairments in the insulin/IGF-1 system and tissue responsiveness, which are determined, among others, by pharmacogenetic factors (see also [53, 63]). This can be true as well for antiaging applications where, in particular, the evaluation of mTOR-related pathways and gene-expression markers is recommended as a means for the choice of geroprotectors [77, 94]. With all the known benefits of metformin in different areas, including reducing the rate of certain complications in diabetic patients [67, 68], only further studies will allow the specific molecular targets of metformin to be elucidated fully with respect to treatment and prevention of obesity, cancer, other age-related pathology and lifespan as discussed above.

Of special interest is the question of whether metformin is the most appropriate biguanide for oncology [36, 53, 95] and beyond. The authors of publications cited herein, while recognizing that only metformin is authorized by currently valid pharmacopeias for clinical use, note that phenformin, which is more associated with lactic acidosis in diabetic patients, may be more potent in anticancer applications, as follows from several preclinical studies [95, 96]. The potential antiaging activity of phenformin was reported long ago [97]. Therefore, it makes sense to compare directly metformin and phenformin for their ability to influence lifespan under identical experimental settings and *in the same experiment* and thus to make an additional step to developing of approaches to the “…recommendation for healthy life, which may help to bring one's lifespan several years closer to the reliably recorded maximum” [98].

## References

[R1] Tannenbaum A, Silverstone H (1953). Nutrition in relation to cancer. Adv Cancer Res.

[R2] Lew EA, Garfinkel L (1979). Variations in mortality by weight among 750000 men and women. J. Chronic Dis.

[R3] Dilman V.M (1994). Development, Aging and Disease. A New Rationale for an Intervention Strategy. Chur: Harwood Academic Publ.

[R4] Calle E.E, Kaaks R (2004). Overweight, obesity and cancer: epidemiological evidence and proposed mechanisms. Nature Reviews Cancer.

[R5] Giovannucci E, Harlan D.M, Archer MC, Bergenstal RM, Gapstur SM, Habel LA, Pollak M, Regensteiner JG, Yee D (2010). Diabetes and cancer: a consensus report. Diabetes Care.

[R6] (2004). Preventing cancer, cardiovascular disease, and diabetes: a common agenda for the American Cancer Society, the American Diabetes Association, and the American Heart Association. CA Cancer J. Clin.

[R7] Blagosklonny MV (2009). Validation of anti-aging drugs by treating age-related diseases. Aging (Albany NY).

[R8] Lugaro G, Giannattasio G, Torti G, Perani G (1967). Studies on the antitumoral activity of the biguanides. V. Arch Ital Patol Clin Tumori.

[R9] Roginsky M.S, Sandler J (1968). Phenformin in human obesity. Ann N Y Acad Sci.

[R10] Dilman VM, Berstein LM, Yevtushenko TP, Tsyrlina YV, Ostroumova MN, Bobrov YuF, Revskoy SYu, Kovalenko IG, Simonov NN (1988). Preliminary evidence on metabolic rehabilitation of cancer patients. Arch Geschwulstforsch.

[R11] Pedersen J (1965). The effect of metformin on weight loss in obesity. Acta endocrinol. (Copenh.).

[R12] Evans JM, Donnelly LA, Emslie-Smith AM, Alessi DR, Morris AD (2005). Metformin and reduced risk of cancer in diabetic patients. BMJ.

[R13] Goodwin PJ, Stambolic V (2011). Obesity and insulin resistance in breast cancer--chemoprevention strategies with a focus on metformin. Breast.

[R14] Dilman VM (1971). Age-associated elevation of hypothalamic, threshold to feedback control, and its role in development, ageing, and disease. Lancet.

[R15] Blagosklonny MV (2011). NCI's provocative questions on cancer: some answers to ignite discussion. Oncotarget.

[R16] Anisimov VN, Sikora E, Pawelec G (2009). Relationships betweencancerandaging: a multilevel approach. Biogerontolo-gy.

[R17] Becker S, Dossus L, Kaaks R (2009). Obesity related hyperinsulinaemia and hyperglycaemia and cancer development. Arch Physiol Biochem.

[R18] Levri K.M, Slaymaker E, Last A, Yeh J, Ference J, D'Amico F, Wilson SA (2005). Metformin as Treatment for Overweight and Obese Adults: A Systematic Review. Ann Fam Med.

[R19] Leblanc E.S, O'Connor E, Whitlock E.P, Patnode CD, Kapka T (2011). Effectiveness of primary care-relevant treatments for obesity in adults: a systematic evidence review for the U.S. Preventive Services Task Force. Ann Intern Med.

[R20] Salpeter SR, Buckley NS, Kahn JA, Salpeter EE (2008). Meta-analysis: metformin treatment in persons at risk for diabetes mellitus. Am J Med.

[R21] Meneghini LF, Orozco-Beltran D, Khunti K, Caputo S, Damçi T, Liebl A, Ross SA (2011). Weight beneficial treatments for type 2 diabetes. J. Clin. Endocrinol. Metab.

[R22] Bruno RV, de Avila MA, Neves FB, Nardi AE, Crespo CM, Sobrinho AT (2007). Comparison of two doses of metformin (2.5 and 1.5 g/day) for the treatment of polycystic ovary syndrome and their effect on body mass index and waist circumference. Fertil Steril.

[R23] Nieuwenhuis-Ruifrok AE, Kuchenbecker WK, Hoek A, Middleton P, Norman RJ (2009). Insulin sensitizing drugs for weight loss in women of reproductive age who are overweight orobese: systematic review andmeta-analysis. Hum Reprod Update.

[R24] Yanovski JA, Krakoff J, Salaita CG, McDuffie JR, Kozlosky M, Sebring NG, Reynolds JC, Brady SM, Calis KA (2011). Effects of metformin on body weight and body composition in obese insulin-resistant children: a randomized clinical trial. Diabetes.

[R25] Srinivasan S, Ambler GR, Baur LA, Garnett SP, Tepsa M, Yap F, Ward GM, Cowell CT (2006). Randomized, controlled trial of metformin for obesity and insulin resistance in children and adolescents: improvement in body composition and fasting insulin. J Clin Endocrinol Metab.

[R26] Moreno-Navarrete J.M, Ortega F.J, Rodríguez-Hermosa J.I, Sabater M, Pardo G, Ricart W, Fernández-Real JM (2011). OCT1 expression in adipocytes could contribute to increased metformin action in obese subjects. Diabetes.

[R27] Delahanty LM, Pan Q, Jablonski KA, Watson KE, McCaffery JM, Shuldiner A, Kahn SE, Knowler WC, Florez JC, Franks PW, for the Diabetes Prevention Program Research Group (2011). Genetic Predictors of Weight Loss and Weight Regain After Intensive Lifestyle Modification, Metformin Treatment, or Standard Care in the Diabetes Prevention Program. Diabetes Care.

[R28] Vincent HK, Taylor AG (2006). Biomarkers and potential mechanisms of obesity-induced oxidantstressin humans. Int J Obes (Lond).

[R29] Chakraborty A, Chowdhury S, Bhattacharyya M (2011). Effect of metformin on oxidative stress, nitrosative stress and inflammatory biomarkers in type 2 diabetes patients. Diabetes Res Clin Pract.

[R30] Anedda A, Rial E, González-Barroso MM (2008). Metformininducesoxidative stressin white adipocytes and raises uncoupling protein 2 levels. J Endocrinol.

[R31] Karelis AD, St-Pierre DH, Conus F, Rabasa-Lhoret R, Poehlman ET (2004). Metabolic and body composition factors in subgroups of obesity: what do we know?. J Clin Endocrinol Metab.

[R32] Shin MJ, Hyun YJ, Kim OY, Kim JY, Jang Y, Lee JH (2006). Weight loss effect on inflammation and LDL oxidation in metabolically healthy but obese (MHO) individuals: low inflammation and LDL oxidation in MHO women. Int J Obes (Lond).

[R33] Després JP (2003). Potential contribution ofmetforminto the management of cardiovascular disease risk in patients with abdominal obesity, the metabolic syndrome and type 2 diabetes. Diabetes Metab.

[R34] Nobes JP, Langley SE, Klopper T, Russell-Jones D, Laing RW (2011). A prospective, randomized pilot study evaluating the effects of metformin and lifestyle intervention on patients with prostate cancer receiving androgen deprivation therapy. BJU Int.

[R35] Bo S, Ciccone G, Rosato R, Villois P, Appendino G, Ghigo E, Grassi G (2011). Cancer mortality reduction and metformin. A retrospective cohort study in type 2 diabetic patients. Diabetes Obes Metab.

[R36] Berstein L.M (2010). Biguanides: An expansion to Practical Oncology (past and present). St.Petersburg, Aesculap.

[R37] Zhang ZJ, Zheng ZJ, Kan H, Song Y, Cui W, Zhao G, Kip KE (2011). Reduced risk of colorectal cancer with metformin therapy in patients with type 2 diabetes: a meta-analysis. Diabetes Care.

[R38] Bodmer M, Becker C, Meier C, Jick SS, Meier CR (2012). Use of Metformin Is Not Associated with a Decreased Risk of Colorectal Cancer: A Case-Control Analysis. Cancer Epidemiol Biomarkers Prev.

[R39] Azoulay L, Dell'Aniello S, Gagnon B, Pollak M, Suissa S (2011). Metformin and the incidence of prostate cancer in patients with type 2 diabetes. Cancer Epidemiol Biomarkers Prev.

[R40] Bosco JL, Antonsen S, Sørensen HT, Pedersen L, Lash TL (2011). Metforminand incidentbreast canceramong diabetic women: a population-based case-control study in Denmark. Cancer Epidemiol Biomarkers Prev.

[R41] Currie CJ, Poole CD, Gale EA (2009). The influence of glucose-lowering therapies on cancer risk in type 2 diabetes. Diabetologia.

[R42] Oliveria SA, Koro CE, Yood MU, Sowell M (2008). Cancer incidence among patients treated with antidiabetic pharmacotherapy. Diabetes Metabol. Syndrome: Clin. Res. & Reviews.

[R43] Lee MS, Hsu CC, Wahlqvist ML, Tsai HN, Chang YH, Huang YC (2011). Type 2 diabetes increases andmetforminreduces total, colorectal, liver and pancreatic cancer incidences in Taiwanese: a representative population prospective cohort study of 800,000 individuals. BMC Cancer.

[R44] Stevens R, McLellan J, Cairns B, Ali R, Bankhead C, Bethel A, Crowe F, Farmer A, Harrison S, Hirst J, Perera R, Pluddemann A, Rose P, Home P, Kahn S, METFORMIN TRIALLISTS' COLLABORATION (2011). Cancer and all-cause mortality: meta-analysis of randomized clinical trials of metformin. Amer. Diab. Assoc. (ADA) Meeting.

[R45] Wild SH (2011). Diabetes, treatments fordiabetesand their effect oncancerincidence and mortality: attempts to disentangle the web of associations. Diabetologia.

[R46] Home PD, Kahn SE, Jones NP, Noronha D, Beck-Nielsen H, Viberti G (2010). ADOPT Study Group; RECORD Steering Committee. Experience of malignancies with oral glucose-lowering drugs in the randomised controlled ADOPT (ADiabetes Outcome Progression Trial) and RECORD (Rosiglitazone Evaluated for Cardiovascular Outcomes and Regulation of Glycaemia in Diabetes) clinical trials. Diabetologia.

[R47] Ruiter R, Visser LE, van Herk-Sukel MP, Coebergh JW, Haak HR, Geelhoed-Duijvestijn PH, Straus SM, Herings RM, Stricker BH (2012). Lower Risk of Cancerin Patients on Metformin in Comparison With Those on Sulfonylurea Derivatives: Results from a large population-based follow-up study. Diabetes Care.

[R48] Bayraktar S, Hernadez-Aya LF, Lei X, Meric-Bernstam F, Litton JK, Hsu L, Hortobagyi GN, Gonzalez-Angulo AM (2011). Effect of metformin on survival outcomes in diabetic patients with triple receptor-negative breast cancer. Cancer.

[R49] Patel T, Kirby W, Hruby G, Benson MC, McKiernan JM, Badani K (2010). Clinical outcomes after radical prostatectomy in diabetic patients treated with metformin. Urology.

[R50] Currie CJ, Poole CD, Jenkins-Jones S, Gale EA, Johnson JA, Morgan CL (2012). Mortality After Incident Cancer in People With and Without Type 2 Diabetes: Impact of metformin on survival. Diabetes Care.

[R51] Lee JH, Kim TI, Jeon SM, Hong SP, Cheon JH, Kim WH (2011). The effects ofmetforminon the survival of colorectalcancerpatients with diabetes mellitus. Int J Cancer.

[R52] Romero IL, McCormick A, McEwen KA, Park S, Karrison T, Yamada SD, Pannain S, Lengyel E (2012). Relationship of Type II Diabetes and Metformin Use to Ovarian Cancer Progression, Survival, and Chemosensitivity. Obstet Gynecol.

[R53] Pollak M (2010). Metformin and otherbiguanidesin oncology: advancing the research agenda. Cancer Prev Res (Phila).

[R54] Dilman VM, Berstein LM, Ostroumova MN, Fedorov SN, Poroshina TE, Tsyrlina EV, Buslaeva VP, Semiglazov VF, Seleznev IK, Bobrov YuF, Vasilyeva IA, Kondratjev VB, Nemirovsky VS, Nikiforov YF (1982). Metabolic immunodepres-sion and metabolic immunotherapy: an attempt of improvement in immunologic response in breast cancer patients by correction of metabolic disturbances. Oncology.

[R55] Berstein LM (2010). Modern approach to metabolic rehabilitation of cancer patients: biguanides (phenformin and metformin) and beyond. Future Oncol.

[R56] Hadad S, Iwamoto T, Jordan L, Purdie C, Bray S, Baker L, Jellema G, Deharo S, Hardie DG, Pusztai L, Moulder-Thompson S, Dewar JA, Thompson AM (2011). Evidence for biological effects of metformin in operable breast cancer: a pre-operative, window-of-opportunity, randomized trial. Breast Cancer Res Treat.

[R57] Hosono K, Endo H, Takahashi H, Sugiyama M, Sakai E, Uchiyama T, Suzuki K, Iida H, Sakamoto Y, Yoneda K, Koide T, Tokoro C, Abe Y, Inamori M, Nakagama H, Nakajima A (2010). Metforminsuppresses colorectal aberrant crypt foci in a short-term clinical trial. Cancer Prev Res (Phila).

[R58] Bershtein L.M, Maximov S.Ya, Danilova M.A, Gershfeld E.D, Boyarkina M.P, Hadzhimba A.S, Kovalevskiy A.Yu, Turkevich E.A, Meshkova I.E (2011). Comparison of aromatase and metformin neoadjuvant effects in endometrial cancer patients. Vopr. Onkol.

[R59] (2012). Editorial. Cancer prevention with a diabetes pill?. Science.

[R60] Niehr F, von Euw E, Attar N, Guo D, Matsunaga D, Sazegar H, Ng C, Glaspy JA, Recio JA, Lo RS, Mischel PS, Comin-Anduix B, Ribas A (2011). Combination therapy with vemurafenib (PLX4032/RG7204) and metformin in melanoma cell lines with distinct driver mutations. J Transl Med.

[R61] Zhuang Y, Miskimins WK (2011). Metformin induces both caspase-dependent and poly(ADP-ribose) polymerase-dependent cell death in breast cancer cells. Mol Cancer Res.

[R62] Shikata E, Yamamoto R, Takane H, Shigemasa C, Ikeda T, Otsubo K, Ieiri I (2007). Human organic cation transporter (OCT1 and OCT2) gene polymorphisms and therapeutic effects of metformin. J Hum Genet.

[R63] Christensen MM, Brasch-Andersen C, Green H, Nielsen F, Damkier P, Beck-Nielsen H, Brosen K (2011). The pharmacogenetics of metformin and its impact on plasma metforminsteady-state levels and glycosylated hemoglobin A1c. Pharmacogenet Genomics.

[R64] Zhou K, Bellenguez C, Spencer CC, Bennett AJ, Coleman RL, Tavendale R, Hawley SA, Donnelly LA, Schofield C, Groves CJ, Burch L, Carr F, Strange A, Freeman C, GoDARTS and UKPDS Diabetes Pharmacogene-tics Study Group; Wellcome Trust Case Control Consortium 2 (2011). Common variants near ATM are associated with glycemic response to metformin in type 2 diabetes. Nat Genet.

[R65] Abbatecola AM, Paolisso G, Corsonello A, Bustacchini S, Lattanzio F (2009). Antidiabetic oral treatment in older people: does frailty matter?. Drugs Aging.

[R66] Salpeter SR, Greyber E, Pasternak GA, Salpeter EE (2010). Risk of fatal and nonfatal lactic acidosis with metformin use in type 2 diabetes mellitus. Cochrane Database Syst Rev.

[R67] Holman R (2007). Metformin as first choice in oral diabetes treatment: the UKPDS experience. Journ. Annu Diabetol Hotel Dieu.

[R68] Tzoulaki I, Molokhia M, Curcin V, Little MP, Millett CJ, Ng A, Hughes RI, Khunti K, Wilkins MR, Majeed A, Elliott P (2009). Risk of cardiovascular disease and all cause mortality among patients with type 2 diabetes prescribed oral antidiabetes drugs: retrospective cohort study using UK general practice research database. BMJ.

[R69] Blagosklonny MV, Campisi J (2008). Cancer and aging: more puzzles, more promises?. Cell Cycle.

[R70] Anisimov VN (2010). Metformin for aging and cancer prevention. Aging (Albany NY).

[R71] Bulterijs S (2011). Metformin as a geroprotector. Rejuvenation Res.

[R72] Dilman VM (1978). Ageing, metabolic immunodepression and carcinogenesis. Mech Ageing Dev.

[R73] Menendez JA, Cufí S, Oliveras-Ferraros C, Martin-Castillo B, Joven J, Vellon L, Vazquez-Martin A (2011). Metformin and the ATM DNA damage response (DDR): accelerating the onset of stress-induced senescence to boost protection against cancer. Aging (Albany NY).

[R74] Oliveras-Ferraros C, Vazquez-Martin A, Menendez JA (2010). Pharmacological mimicking of caloric restriction elicits epigenetic reprogramming of differentiated cells to stem-like self-renewal states. Rejuvenation Res.

[R75] Arkad'eva AV, Mamonov AA, Popovich IG, Anisimov VN, Mikhel'son VM, Spivak IM (2011). Metformin slows down ageing processes at the cellular level in SHR mice. Tsitologiia.

[R76] Halicka HD, Zhao H, Li J, Traganos F, Zhang S, Lee M, Darzynkiewicz Z (2011). Genome protective effect of metformin as revealed by reduced level of constitutive DNA damage signaling. Aging (Albany NY).

[R77] Blagosklonny MV (2011). Molecular damage in cancer: an argument for mTOR-driven aging. Aging (Albany NY).

[R78] Onken B, Driscoll M (2010). Metformin Induces a Dietary Restriction-Like State and the Oxidative Stress Response to Extend *C. elegans* Healthspan via AMPK, LKB1, and SKN-1. PLoS One.

[R79] Ma TC, Buescher JL, Oatis B, Funk JA, Nash AJ, Carrier RL, Hoyt KR (2007). Metformin therapy in a transgenic mouse model of Huntington's disease. Neurosci Lett.

[R80] Spindler SR (2012). Review of the literature and suggestions for the design of rodent survival studies for the identification of compounds that increase health and life span. Age (Dordr).

[R81] Pianka E.R (1970). On r and K selection. American Naturalist.

[R82] Golubev AG (2003). Biochemistry of lifespan extension. Adv Gerontol.

[R83] Anisimov VN, Berstein LM, Egormin PA, Piskunova TS, Popovich IG, Zabezhinski MA, Kovalenko IG, Poroshina TE, Semenchenko AV, Provinciali M, Re F, Franceschi C (2005). Effect ofmetforminonlifespanand on the development of spontaneous mammary tumors in HER-2/neu transgenic mice. Exp Gerontol.

[R84] Anisimov VN, Egormin PA, Piskunova TS, Popovich IG, Tyndyk ML, Yurova MN, Zabezhinski MA, Anikin IV, Karkach AS, Romanyukha AA (2010). Metformin extends life span of HER-2/neu transgenic mice and in combination with melatonin inhibits growth of transplantable tumors in vivo. Cell Cycle.

[R85] Anisimov VN, Berstein LM, Egormin PA, Piskunova TS, Popovich IG, Zabezhinski MA, Tyndyk ML, Yurova MV, Kovalenko IG, Poroshina TE, Semenchenko AV (2008). Metforminslows down aging and extendslifespanof female SHR mice. Cell Cycle.

[R86] Yurova M.N (2011). Effect of plastochinone derivative on spontaneous carcinogenesis and life span in mice. Candidate Thesis.

[R87] Anisimov VN, Piskunova TS, Popovich IG, Zabezhinski MA, Tyndyk ML, Egormin PA, Yurova MV, Rosenfeld SV, Semenchenko AV, Kovalenko IG, Poroshina TE, Berstein LM (2010). Gender differences in metformin effect on aging, life span and spontaneous tumorigenesis in 129/Sv mice. Aging (Albany NY).

[R88] Anisimov VN, Berstein LM, Popovich IG, Zabezhinski MA, Egormin PA, Piskunova TS, Semenchenko AV, Tyndyk ML, Yurova MN, Kovalenko IG, Poroshina TE (2011). If started early in life, metformin treatment increases life span and postpones tumors in female SHR mice. Aging (Albany NY).

[R89] Smith DLJr, Elam CF, Mattison JA, Lane MA, Roth GS, Ingram DK, Allison DB (2010). Metforminsupplementation and life span in Fischer-344 rats. J Gerontol A Biol Sci Med Sci.

[R90] Blagosklonny MV (2010). Metformin and sex: Why suppression of aging may be harmful to young male mice. Aging (Albany NY).

[R91] Berstein LM (1988). Newborn macrosomyand cancer. Adv Cancer Res.

[R92] Mercken EM, Carboneau BA, Krzysik-Walker SM, de Cabo R (2011). Of mice and men: The benefits of caloric restriction, exercise, and mimetics. Ageing Res Rev.

[R93] Smith DL, Nagy TR, Allison DB (2010). Calorie restriction: what recent results suggest for the future of ageing research. Eur J Clin Invest.

[R94] Spindler SR, Mote PL (2007). Screening candidate longevity therapeutics using gene-expression arrays. Gerontology.

[R95] Chong CR, Chabner BA (2009). Mysteriousmetformin. Oncologist.

[R96] Segal ED, Yasmeen A, Beauchamp MC, Rosenblatt J, Pollak M, Gotlieb WH (2011). Relevance of the OCT1 transporter to the antineoplastic effect of biguanides. Biochem Biophys Res Commun.

[R97] Dilman VM, Anisimov VN (1980). Effect of treatment with phenformin, diphenylhydantoin or L-dopa on life span and tumour incidence in C3H/Sn mice. Gerontology.

[R98] Golubev AG (2009). The issue of feasibility of a general theory of aging. III. The theory and practice of aging. Adv Gerontol.

